# Anti-HBV Activities of Polysaccharides from *Thais clavigera* (Küster) by In Vitro and In Vivo Study

**DOI:** 10.3390/md19040195

**Published:** 2021-03-30

**Authors:** Fei Tang, Guanghua Huang, Liping Lin, Hong Yin, Lili Shao, Ruian Xu, Xiuling Cui

**Affiliations:** 1 Engineering Research Center of Molecular Medicine, Ministry of Education, School of Medicine, Huaqiao University, Xiamen 361021, China; tangfeihn@163.com (F.T.); Huangguanghua0818@163.com (G.H.); linliping.yichen@foxmail.com (L.L.); ccy15915834141@163.com (H.Y.); shaolili517@163.com (L.S.); 2 Fujian Key Laboratory of Molecular Medicine, Key Laboratory of Precision Medicine and Molecular Diagnosis of Fujian Universities, Xiamen Key Laboratory of Marine and Gene Drugs, Xiamen 361021, China

**Keywords:** *Thais clavigera* (Küster), polysaccharides, anti-HBV activities, in vitro, in vivo

## Abstract

Hepatitis B virus (HBV) infection remains a major global health problem. It is therefore imperative to develop drugs for anti-hepatitis B with high-efficiency and low toxicity. Attracted by the observations and evidence that the symptoms of some patients from the Southern Fujian, China, suffering from hepatitis B were alleviated after daily eating an edible marine mollusk, *Thais clavigera* (Küster 1860) (TCK). Water-soluble polysaccharide from TCK (TCKP1) was isolated and characterized. The anti-HBV activity of TCKP1 and its regulatory pathway were investigated on both HepG2.2.15 cell line and HBV transgenic mice. The data obtained from in vitro studies showed that TCKP1 significantly enhanced the production of IFN-α, and reduced the level of HBV antigens and HBV DNA in the supernatants of HepG2.2.15 cells in a dose-dependent manner with low cytotoxicity. The result of the study on the HBV transgenic mice further revealed that TCKP1 significantly decreased the level of transaminases, HBsAg, HBeAg, and HBV DNA in the serum, as well as HBsAg, HBeAg, HBV DNA, and HBV RNA in the liver of HBV transgenic (HBV-Tg) mice. Furthermore, TCKP1 exhibited equivalent inhibitory effect with the positive control tenofovir alafenamide (TAF) on the markers above except for HBV DNA even in low dosage in a mouse model. However, the TCKP1 high-dose group displayed stronger inhibition of transaminases and liver HBsAg, HBeAg, and HBV RNA when compared with those of TAF. Meanwhile, inflammation of the liver was, by pathological observation, relieved in a dose-dependent manner after being treated with TCKP1. In addition, elevated levels of interleukin-12 (IL-12) and interferon γ (IFN-γ), and reduced level of interleukin-4 (IL-4) in the serum were observed, indicating that the anti-HBV effect of TCKP1 was achieved by potentiating immunocyte function and regulating the balance of Th1/Th2 cytokines.

## 1. Introduction

Despite the popularization of a safe and effective vaccine against hepatitis B virus (HBV), HBV infection remains a major global health problem with 257 million people being chronically infected worldwide [1]. One-third of these carriers are projected to develop severe end-stage liver diseases including liver failure, cirrhosis, and hepatocellular carcinoma (HCC), and over 887,000 people die from these liver diseases caused by HBV infection per year [2,3,4]. There are some anti-HBV drugs available today, such as interferon-α (IFN-α), the nucleoside analogues, e.g., lamivudine (3TC), adefovir dipivoxil, and tenofovir alafenamide (TAF). However, great challenges still remain—for instance, limited effectiveness (only 40% response rate), expensive cost, and frequent and serious adverse effects experienced by patients through interferon-α therapy [5]. Nevertheless, significant issues exist with nucleoside analogues, such as moderate efficiency, dose-dependent side effects, and drug resistance [6]. Polysaccharides, as complicated polymers, have been reported to have comprehensive bioactivities, including anti-oxidation, anti-tumor, anti-viral, anti-bacterial, hypoglycemic activity, immunoregulation, and hepatic protective activities [7,8,9,10,11,12]. In recent years, many polysaccharides from plants have been shown to possess excellent anti-HBV activities [13,14]. Only a few polysaccharides from marine creatures, polyguluronate sulfate, scallop polysaccharide, for instance, have been found with considerable anti-HBV activity on HepG2.2.15 cells [15,16,17,18]. However, the precise function and mechanism of these polysaccharides in anti-HBV activity in vivo are still unknown.

*Thais clavigera* (Küster 1860) (TCK), also recognized as *Reishia clavigera* (Küster 1860), is a popular edible conch widely distributed in the Southeast Asia, Australia, the Gulf of Mexico, and other regions [19]. TCK could be used for marine water pollution monitoring, due to the sensitivity of its reproductive system to water contamination [20]. It has been reported that an extraction of TCK has obvious anti-inflammatory activity [21]. The significant liver protection of TCK from alcoholic liver and hepatitis B, has been confirmed by a large number of anecdotal stories in Southern Fujian, China. Attracted by observations that the symptoms of some patients suffering from hepatitis B were reduced after having eaten TCK daily, we have carried out a set of preliminary and systematical studies over the past decade. Initial investigations showed that the water extraction of TCK exhibited pronounced anti-HBV activity, but the ingredients are still unclear.

The aim of this study was to investigate the inhibitory effects of the polysaccharide TCKP1 isolated and purified from TCK against HBV both in vitro and in vivo. Firstly, TCKP1 was prepared and purified and characterized by constituent analysis. Then the effects of TCKP1 on the production of HBV surface antigen (HBsAg), HBV e antigen (HBeAg), and HBV DNA copies in supernatant of HepG2.2.15 cells were evaluated. Finally, TCKP1 and a positive control (tenofovir alafenamide, TAF) were tested on HBV transgenic (HBV-Tg) mice to determine its anti-HBV activity in vivo and the underlying mechanism.

## 2. Results

### 2.1. Characterization of TCKP1

**Figure 1:**
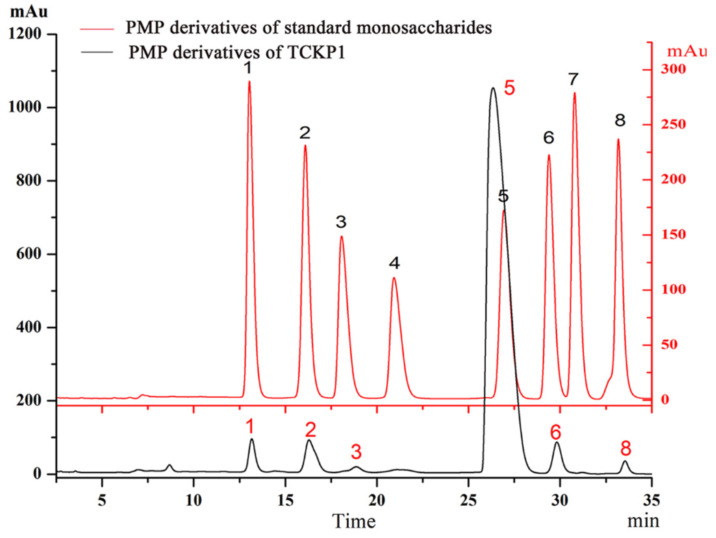
Comparison of 1-phenyl-3-methyl-5-pyrazolone (PMP) derivatives of standard monosaccharide and water-soluble polysaccharides from *Thais clavigera* (Küster 1860) (TCKP1) via the HPLC method. 1, mannose (Man); 2, glucosamine (GlcN); 3, glucuronic acid (GlcA); 4, GalA; 5, glucose (Glc); 6, galactose (Gal); 7, xylose (Xyl); and 8, arabinose (Ara).

The water-soluble polysaccharide TCKP1 was obtained with a yield of 4.27%, including 71.2% total sugar, 9.3% sulfate, and 1.27% protein. In addition, 0.79% overall glucuronic acid (GlcA) was detected through carbazole reaction. The monosaccharide composition of TCKP1 was analyzed by HPLC after hydrolyzation and derivatization with 1-phenyl-3-methyl-5-pyrazolone (PMP). As shown in Figure 1, TCKP1 is composed of mannose (Man), glucosamine (GlcN), glucuronic acid (GlcA), glucose (Glc), galactose (Gal), and arabinose (Ara) in a molar ratio of 9:16:4:512:9:4 (Table 1).

**Figure 2:**
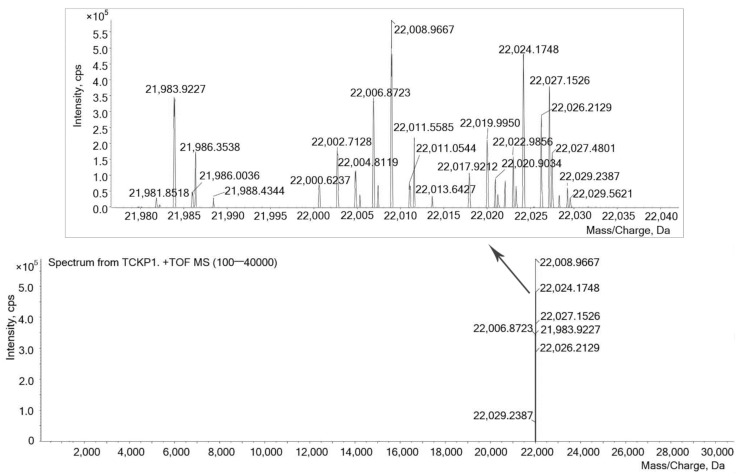
ESI-MS spectrum of TCKP1. Conditions: 100 μg/mL TCKP1 with methanoic acid. A triple TOF mass spectrometer was used (100–40,000 Da).

Molecular weight distribution of TCKP1 ranges from 21,981.8 to 22,029.2 Da, which was measured by ESI-TOF-MS, and the range of mass/charge was set at 100 to 40 kDa, as shown in Figure 2. Signals at 22,011.5585 and 22,011.0544 Da indicated the existence of polysaccharide dimer with two electric charges (MW = 44,021 Da), while signals at 22,027.1526 and 22,027.4801 Da demonstrated the existence of polysaccharide trimer with three electric charges (MW = 66,078 Da).

### 2.2. Effects of Treatment with TCKP1 on HepG2.2.15 Cells

**Figure 3:**
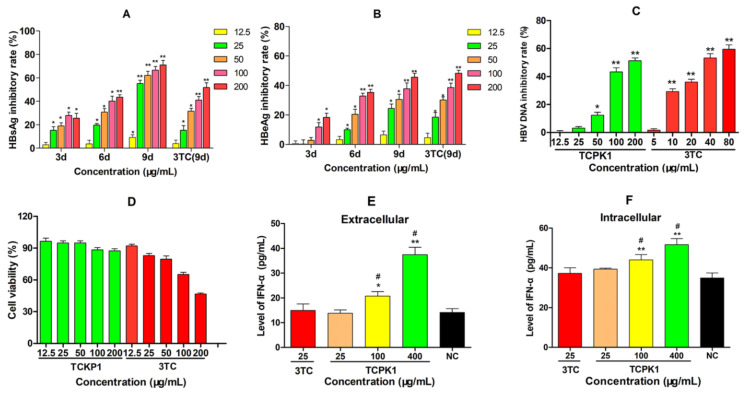
Inhibitory effect of TCPK1 on HBsAg (**A**), HBeAg (**B**), and HBV DNA (**C**) in the supernatants of HepG2.2.15 cells and the effect on cell viability (**D**). Lamivudine (3TC) treated for 9 days was used as the positive control in A and B, and for 6 days in C. The promoting effect of TCKP1 on the level of interferon-α (IFN-α) in the culture supernatants (extracellular, (**E**)) and the cell lysates (intracellular, (**F**)) of HepG2.2.15 cells after the treatment for 3 days with 25 μg/mL lamivudine (3TC) as the positive control. All values are expressed as means ± S.D. from three separate experiments performed in triplicate. * *p* < 0.05, ** *p* < 0.01 vs. the negative control. ^#^ *p* < 0.05 vs. the 3TC positive control.

The anti-HBV ability of TCPK1 to inhibit the production of HBsAg, HBeAg, and HBV DNA was evaluated by the 50% cytotoxicity concentration (CC_50_) and 50% inhibition concentration (IC_50_) [22]. As shown in Figure 3, the levels of antigens in the supernatant of HepG2.2.15 cells were reduced by TCKP1 and 3TC in a dose- and time-dependent manner. The inhibitory effect of TCKP1 on HBsAg was stronger than that of 3TC, while its effect on HBeAg was similar to 3TC at the same concentration. The IC_50_ values of TCKP1 in HBsAg and HBeAg were 43.7 μg/mL, 212.6 μg/mL, respectively, and the IC_50_ values of 3TC on HBsAg and HBeAg were 154.6 μg/mL and 181.9 μg/mL. Generally, DNA copies are regarded as one of the most important indicators for evaluating anti-HBV activity of drugs. The inhibitory effect of TCPK1 on HBV DNA synthesis was fairly obvious (IC_50_ = 160.2 μg/mL), but was weaker than that of 3TC (IC_50_ = 40.5 μg/mL) after the HepG2.2.15 cells treated with TCPK1 for 6 days (Figure 3C). On the contrary, the HepG2.2.15 cells treated with TCPK1 retained low cytotoxicity even at the concentration of 200 μg/mL (about 87.5% cell viability rate), i.e., TCPK1 possessed much higher security (CC_50_ >> 200 μg/mL, Figure 3D) when compared with 3TC (CC_50_ = 193.6 μg/mL), detected by CCK-8 kit.

### 2.3. Effects of TCKP1 on the Level of IFN-α in HepG2.2.15 Cells

The changes in IFN-α level in HepG2.2.15 cells were observed after treatment with TCKP1 for 3 days to assess the immunocyte function of TCKP1 in vitro. The level of IFN-α in the culture supernatants and cell lysates treated with TCKP1 (100 and 400 μg/mL) both significantly increased with dose (*p* < 0.05 or 0.01). However, such changes were not observed in the positive control of 3TC (Figure 3E,F). These data implied that anti-HBV effect of TCKP1 on HepG2.2.15 cells was achieved through enhancing the interferon system.

### 2.4. Effects of Treatment with TCKP1 on HBV Transgenic Mice

#### 2.4.1. Level of HBV DNA, HBsAg, and HBeAg in Serum

**Figure 4:**
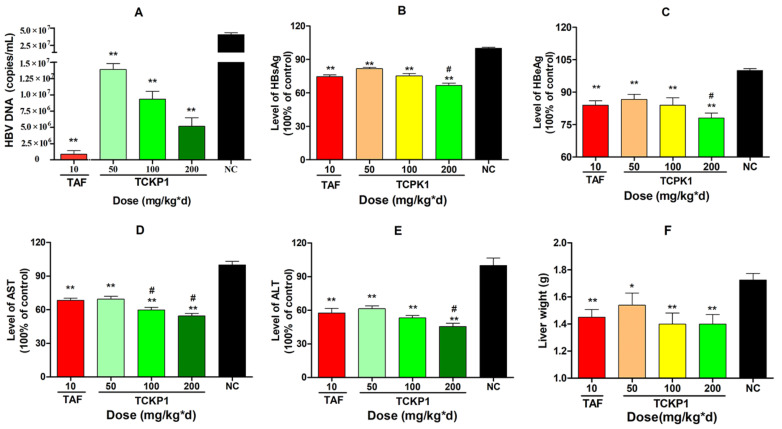
The level of DNA (**A**) and inhibitory effect of TCPK1 on HBsAg (**B**), HBeAg (**C**), AST (**D**), and ALT (**E**) in the serum of transgenic HBV mice. The effect of TCKP1 on liver weight of transgenic HBV mice (**F**). All values are expressed as means ± S.D. (*n* = 5). * *p* < 0.05, ** *p* < 0.01 vs. the negative control (NC); ^#^ *p* < 0.05 vs. the positive control (tenofovir alafenamide, TAF).

After HBV-Tg mice were treated for 28 days intra-peritoneally, HBV DNA levels in the TCKP1 low- (50 mg/kg·d), medium- (100 mg/kg·d), and high- (200 mg/kg·d) dose groups and the TAF group were significantly decreased by 65.7%, 77.0%, 87.2%, and 99.6%, respectively, when compared with the negative control (NC) group (*p* < 0.01). The results showed that the inhibition activity of TCKP1 on HBV DNA synthesis in vivo increased with dose, but was slightly lower than that of TAF (Figure 4A).

Level of serum HBsAg and HBeAg in all treated groups significantly reduced in a dose-dependent manner when compared with the NC group (*p* < 0.01). Correspondingly, the data showed that inhibition of HBsAg was much stronger than that of HBeAg (Figure 4B,C), and there was no significant difference between the TCKP1-low-dose group and the TAF group in the level of antigens (*p* > 0.05), while the inhibitory effect of HBsAg of high-dose TCKP1 was even stronger than that of TAF (*p* < 0.05).

#### 2.4.2. Levels of AST and ALT in Serum

Excessive levels of transaminases, such as aspartate aminotransferase (AST) and alanine aminotransferase (ALT), in serum are generally regarded as the impairment of liver function. After the treatment, there were significant decreases in the aminopherase levels for both the TCKP1 groups and the TAF group (*p* < 0.01 vs. NC group). The TCKP1 low-dose group showed equivalent inhibitory action on ALT and AST levels. Moreover, the level of ALT and AST in the TCKP1 high-dose group, and the level of ALT in the TCKP1 medium-dose group appeared significantly lower than those of TAF group (Figure 4D,E), implying that TCKP1 possesses a powerful hepatic protective function from HBV.

#### 2.4.3. The Effects of TCKP1 on Liver Morphologies, Weight, and Histopathological Examination

Liver wight: As shown in Figure 4F, the liver weight in the NC group was significantly higher than those in the treated groups (*p* < 0.05), indicating that TCKP1 could alleviate the liver swelling caused by HBV. However, there was no significant difference between the TCKP1 groups and the TAF group.

**Figure 5:**
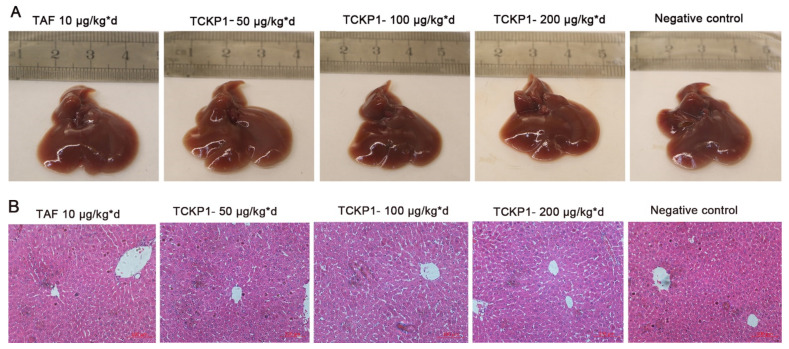
The effect of TCKP1 on liver morphological (**A**) and histopathological (**B**) changes of HBV transgenic mice. Liver tissue sections were subjected to histological examinations by staining with hematoxylin and eosin under a light microscope. Graphs shown are representative images from different groups of mice (H&E staining, ×200) (bar = 100 μm).

Liver morphology: The livers of HBV-Tg mice in the NC group were maroon, with slight swelling, a few needle-like necrotic foci and tubercule on the surface, and plenty of visible vascular congestion on the edge (Figure 5A), while liver congestion and swelling in the TCKP1 groups was improved to varying degrees. These observations reconfirmed that TCKP1 could reduce the impairment of liver function and protect the liver from HBV to a considerable degree.

Histopathological examination of livers: In order to monitor pathological change, H&E staining of liver sections from the various treatment groups were examined under the light microscope after the therapy. The histopathological examination (Figure 5B) visually displayed swollen hepatocytes, hyperchromatic nuclei, disordered hepatic lobule structure, and hardly visible hepatic cords in the livers of the NC group. The liver tissue of the treated groups had an obvious recovery—the swelling of liver cells was distinctly reduced, with part of the hepatic cord and hepatic sinus structure visible. The degree of recovery in the TCKP1 group generally increased with dose. The result further revealed the hypotonicity of TCKP1 even at the dosage of 200 mg/kg·day.

#### 2.4.4. Levels of HBV DNA, HBsAg, HBeAg, and HBV RNA in the Livers of the Experimental Mice

**Figure 6:**
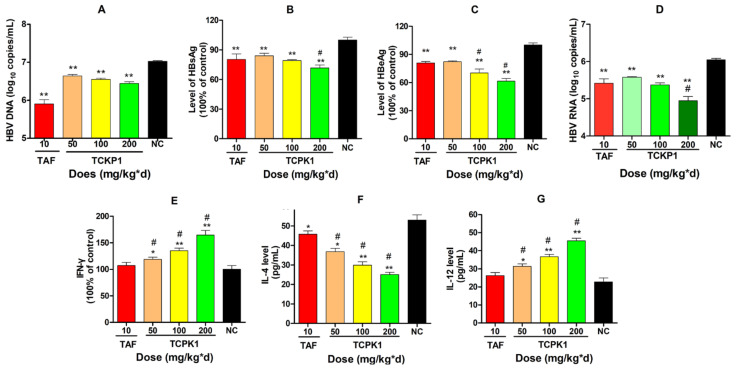
Inhibitory effect of TCPK1 on the production of HBV DNA (**A**), HBsAg (**B**), HBeAg (**C**), and HBV RNA (**D**) in livers of HBV transgenic mice. Effect of TCPK1 on level of IFN-γ (**E**), IL-4 (**F**), and IL-12 (**G**) in the serum of HBV-Tg mice. All values are expressed as means ± S.D. (*n* = 5). * *p* < 0.05, ** *p* < 0.05 vs. the negative control (NC); ^#^ *p* < 0.05 vs. the positive control (tenofovir aAlafenamide, TAF).

Significant dose-response to suppress HBsAg and HBeAg expression and HBV DNA levels were observed in the liver tissues of all treated groups when compared with the negative control (Figure 6A–C). The inhibitory effect of TCKP1 on HBV DNA in all treated groups was weaker than that of TAF. However, the inhibition effect on HBsAg for the TCKP-H group, and inhibition on HBeAg for both TCKP-M and TCKP-H groups were stronger than that of the TAF group (*p* < 0.05).

The level of HBV RNA was also measured to evaluate the expression of HBV DNA in liver tissues after the mice were treated for 28 days (Figure 6D). As a consequence, there was a significant decrease in HBV RNA copies in all treated groups, when compared with the NC (*p* < 0.01). Although the inhibition of DNA synthesis in the TCKP1 group was weaker than that of TAF, the level of HBV RNA in the TCKP1-H group was much lower than that of TAF group (*p* < 0.05), suggesting the mechanism of anti-HBV activity of TCPK1 might differ from that of TAF.

#### 2.4.5. Levels of IFN-γ, IL-4, and IL-12 in the Serum of Experimental Mice

In order to evaluate the effects of TCPK1 on immune response and explore the underlying mechanism, level of interferon γ (IFN-γ), interleukin-4 (IL-4), and interleukin-12 (IL-12) in serum of HBV-Tg mice were assessed by using ELISA kits. As a result, there was a significant increase in IFN-γ and IL-12 levels but a reduction in IL-4 level in TCKP1 groups in a dose-dependent manner when compared with the negative control group (*p* < 0.05 or *p* < 0.01, Figure 6E–G) in the serum of HBV-Tg mice. Nevertheless, there was no significant difference between TAF (10 mg/kg·d) and the negative control group (*p* > 0.05) except for IL-4. The promotion effect of TCKP1 on IFN-γ suggested that the antiviral effect of TCKP1 in HBV-Tg mice was probably associated with the interferon system, while there was an increase in IL-12 and a decrease in IL-4, suggesting that TCKP1 could be involved in regulation of the balance of Th1/Th2 cytokines.

## 3. Discussion

The hepatology investigation has been carried out for over a decade by our group [23,24]. Attracted by the observations that the symptoms of some patients from Southern Fujian, China, suffering from hepatitis B were alleviated after daily eating of the marine mollusk *Thais clavigera* (Küster) (TCK), a popular edible conch from the Southeast China sea, we therefore launched a systematical investigation into the anti-HBV effect of this species.

In this study, the monosaccharide compositions of TCKP1 polysaccharides from marine mollusk TCK (Figure 1) were characterized. TCKP1 was constituted as mannose (Man), glucosamine (GlcN), glucuronic acid (GlcA), glucose (Glc), galactose (Gal), and arabinose (Ara) in a molar ratio of 9:16:4:512:9:4 (Table 1). The ESI-TOF mass spectrum of TCKP1 indicated that TCKP1 is mainly composed of a polysaccharide unit with mass peaks (M + H) + distributed from 21,981.8 to 22,026.2 Da together with dimeric and trimeric polysaccharides.

Moreover, the anti-HBV activity of TCKP1 and interaction mechanisms were explored by both in vitro and in vivo approaches. TCKP1 showed a much stronger inhibition effect on HBsAg and similar inhibition effect on HBeAg when compared to that of 3TC at the same concentration under identical experimental conditions on HepG2.2.15 cells. Obviously, TCKP1 is of more efficient inhibitor of HBsAg (IC_50_ = 43.7 μg/mL, 71.0% inhibition rate at 200 μg/mL), when compared with the majority of other known polysaccharides available in HepG2.2.15 cells. This result can be compared with 51.8% at 250 μg/mL for a polyguluronate sulfate [15], 27.9% for *Saussurea laniceps* polysaccharide [9], 66.7% [25] for *Lentinus edodes* polyguluronate, and 62.5% [26] for polyguluronate from *Radix isatidis* at 200 μg/mL. The superior anti-HBV activity of TCKP1 might be partially associated with the synergistic effect of various polysaccharides, which has been observed in previous work on polysaccharides [27,28]. Moreover, HBV DNA was also significantly decreased in experimental mice treated with TCKP1 (IC_50_ = 160.2 μg/mL), but TCKP1 was weaker than that of 3TC (IC_50_ = 40.5 μg/mL) in its inhibition effect (Figure 3C). Encouragingly, the CC_50_ of TCKP1 was much higher than 200 μg/mL, which means TCKP1 has much wider selective dosage ranges with slight cytotoxicity only (CC_50_ of 3TC is 193.6 μg/mL).

The in vivo experiment reconfirmed that TCKP1 exhibited a potent inhibitory effect in a dose-dependent manner on HBV replication. HBV-Tg mice treated with TCKP1 had significantly reduced levels of serum HBV DNA, HBsAg, HBeAg, transaminases ALT and AST, liver antigens, HBV DNA, and HBV RNA. It would be worth noting that there was no significant different between TCKP1 low-dose (50 mg/kg·d) and the TAF group (10 mg/kg·d) in the level of HBV antigens, ALT, AST, and HBV RNA. It is gratifying that the clearance for serum transaminase, which are closely associated with liver damage, in the TCKP-M and TCKP-H group was much higher than that of the TAF. In other words, TCKP1 clearly improved hepatic impairment in transgenic mice better than TAF at the dose of 100 and 200 mg/kg·d.

In addition, morphology observations and histological examinations on the livers of all experimental mice demonstrated that TCKP1 clearly improved hepatic pathological changes, and TCKP1 is much safer than TAF. Therefore, TCKP1 exhibits outstanding anti-HBV activity in vivo.

Mechanically, it has been reported that the antiviral activity of polysaccharides is achieved by preventing the virus from infecting cells or enhancing the immune system [29,30]. Because of the structural complexity of polysaccharides, the anti-HBV mechanism of most polysaccharides still remains unclear. The only recent report on anti-HBV activity of polysaccharides suggested that the polysaccharide obtained from *Radix isatidis* enhanced the production of IFN-α in HepG2.2.15 cells possibly via activation of the JAK/STAT signal pathway and induction of antiviral proteins [27]. The results of the present study exhibit that the inhibition of HBV RNA synthesis by TCKP1 is much stronger than that of DNA synthesis, indicating that the anti-viral functions and pathway of TCKP1 could be different from that of nucleoside analogs, such as TAF.

IFN therapy has been widely accepted as an anti-HBV drug for the majority of chronic HBV-infected patients. HBV is susceptible to cellular immune responses, especially to the signal of IFN-α, which can reduce of level of HBV protein, RNA, and possibly DNA [31]. Therefore, the effect of TCKP1 on IFN-α production in HepG2.2.15 cells was tested to assess its immunocyte function. Evidence from the results obtained showed that IFN-α in the culture supernatants and cell lysates treated with TCKP1 (100 and 400 μg/mL) for 3 days was both significantly increased with dosage. On the contrary, there was no obvious effect on IFN-α level for cells treated with 3TC (25 μg/mL) (Figure 3E,F). These results suggested that TCKP1 might execute anti-HBV activity in vitro through enhancing the interferon system.

Generally, chronic HBV infection is closely associated with immune dysfunction, for instance, imbalance of Th1/Th2 [32]. Th1 secretes cytokines that induce cellular immune response as IFN-γ, while Th2 secretes cytokines represented by IL-4 to induce humoral immune response [33]. The imbalance of Th1/Th2 usually leads to disorders of cellular and humoral immunity, and eventually results in chronic infection [34]. In addition, IL-12 strongly inhibits the process of replication of HBV and eliminates HBV though inducing the release of endogenous IFN-γ and Th1 cytokines produced by CD4^+^, CD8^+^ T cells, and NK cells [35].

We, therefore, evaluated the effect of TCPK1 on immune response in vivo. The experimental data (Figure 6E–G) of the present study showed that there was a significant increase in IFN-γ and IL-12 levels but a significant reduction in IL-4 level in TCKP1 groups in a dose-dependent manner compared with the negative control group (*p* < 0.05 or *p* < 0.01). However, there was no significant difference between TAF (10 mg/kg·d) and the negative control group (*p* > 0.05) except for IL-4, suggesting that there was a totally different anti-HBV pathway between TCPK1 and TAF. TCPK1 should have its own physiological functions and regulation system for anti HBV recovery. Such differences in pathway and regulation system could well explain why TCKP1 presented a lower HBV DNA inhibition rate but showed similar inhibition of antigens, aminopherase, and HBV RNA in low-dose and stronger inhibition in high-dose, compared with TAF (10 mg/kg·d) (Figure 6A,D). Clinically, the balance of IFN-γ and IL-4 usually reflects the balance of Th1/Th2 cell, which indicates the recovery process of immunity in patients infected with HBV. An increase in levels of IFN-γ and IL-12, and a decrease in levels of IL-4 treated with TCKP1 implies that anti-HBV activity of TCKP1 was achieved by potentiating immunocyte function and regulating the balance of Th1/Th2 cytokines.

In conclusion, the present study, for the first time, demonstrated that TCKP1 displayed significant anti-HBV activity with extremely low toxicity in both HepG2.2.15 cells and the HBV transgenic mouse model though potentiating immunocyte function. Meanwhile, prominent immune responses for HBV injury recovery and low-hepatotoxicity might be two of the best advantages of TCKP1 when compared with strong anti-HBV nucleoside analogues, such as TAF.

Our study supports preclinical evidence for the observations that the symptoms of some patients from Southern Fujian, China, suffering from hepatitis B are alleviated after daily eating of marine mollusk TCK, and demonstrates that TCKP1 has great potential for developing as an anti-hepatitis B medicine.

## 4. Materials and Methods

### 4.1. Chemicals and Reagents

Dulbecco’s modified eagle medium (DMEM), phosphate buffer solution (PBS), and 0.25% of trypsin (with EDTA) were purchased from HyClone (GE Life Sciences, Logan, UT, USA). Fetal bovine serum (FBS) was obtained from Gibco (Thermo Fisher Scientific, Paisley, Renfrewshire, UK). Cell Counting Kit-8 (CCK-8) was purchased from Dojindo (Shanghai, China). The compound 3TC was purchased from Heowns Biochem LLC (Tianjin, China). Tenofovir alafenamide (TAF) was purchased from Shanghai pharmaceutical technology Co., Ltd. (Shanghai, China). G418 were purchased from Beyotime Biotechnology (Shanghai, China). The enzyme-linked immunosorbent assay (ELISA) diagnostic kit for HBV surface antigen (HBsAg) and e antigen (HBeAg) was obtained from Shanghai Kehua Bio-engineering Co., Ltd. (Shanghai, China). HBV DNA extraction and quantitative detection kit (PCR fluorescence probe method) was obtained from Acon Biotech (Hangzhou) Co., Ltd. (Hangzhou, China). ALT and AST kits (microtest) were purchased from Suzhou Comin Biotechnology Co., Ltd. (Suzhou, China). RIPA buffer was purchased from New Cell & Molecular Biotec Co., Ltd. (Suzhou, China). Cell/Tissue Total RNA Isolation Mini Kit and HiScript II Q RT SuperMix for qPCR were purchased from Nanjing Vazyme Biotech Co., Ltd. (Nanjing, China). Mouse IFN-γ ELISA kit was purchased from MSK Biotechnology Co., Ltd. (Wuhan, China). IFN-α, IL-4, and IL-12 ELISA kit was purchased from Nanjing Jiancheng Bioengineering Institution (Nanjing, China). RIPA (radio immunoprecipitation assay) buffer was purchased from New Cell and Molecular Biotech Co., Ltd. (Suzhou, China). Papain was obtained from Nanning Pangbo biological engineering Co., Ltd. (Nanning, China). The standard monosaccharides, namely glucose (Glc), galactose (Gal), glucosamine (GlcN), mannose (Man), xylose (Xyl), arabinose (Ara), glucuronic acid (GlcA), and galacturonic acid (GalA) were purchased from Sinopharma Chemical Reagent Co., Ltd. (Shanghai, China). 1-phenyl-3-methyl-5-pyrazolone (PMP) were purchased from Sigma Company (Saint Louis, MO, USA), All other reagents used in this were analytical grade. Ultrapure water was produced by Milli-Q system (Millipore, Billerica, MA, USA).

### 4.2. Materials and Preparation of TCKP1

Medium sized *Thais clavigera* (Küster) were collected from Jinjiang peninsular, on the Southern coast of Fujian province of China, and were authenticated by the Third Institute of Oceanography, Ministry of Natural Resource, China. The voucher specimens have been deposited in Huaqiao University. The processed snails were kept frozen at −20 °C prior to purification.

Twenty-five kilogram of fresh TCK was boiled for 3–5 min in hot water after washing. The edible parts were dehydrated by vacuum freeze drier at −80 °C for 24 h, and 1868 g dry-out flesh was gained. The harvested dry tissue (300 g) of TCK was pulverized and degreased three times with n-hexane at a solid:liquid ratio of 1:10. After suction filtration and air drying, the TCK powder was extracted twice with distilled water containing 1% NaOH (*w*/*v*) at a ratio of 1:10 for 2 h at 70 °C. Subsequently, the filtrates were gathered and concentrated with a scraped film evaporator (ASZ-2000A, All Herbal Scientech, city, China) at 65 °C. The concentrate was neutralized with HCl, and precipitated by adding four volumes of ethanol and kept at 4 °C for 40 h [36]. The precipitate was collected via centrifugation, and solubilized in ultrapure water with as little volume as possible. Then repeat the alcohol precipitation and centrifugation steps above were repeated twice more, primarily to reduce impurities such as protein and glycoprotein. The crude polysaccharide was deproteinated by enzymolysis with 4% papain (*w*/*w*) at pH = 7.0, 60 °C for 2 h, and was incubated at 90 °C for 15 min to terminate the enzymolysis. Then the hydrolysate was deproteinated again using the Sevag method 20 times, until there was no obvious precipitation.

The deproteinized crude polysaccharide was concentrated after depigmented with macroporous resin D101. In order to remove all of the small molecules (monosaccharides, salts or oligosaccharides, for instance), the polysaccharides were centrifuged using 3 kD and 10 kD ultrafiltration tubes. The supernatant in the 10 kD ultrafiltration tubes was concentrated and freeze-dried to give TCKP1 (12.8 g), with a yield of 4.27%. The chemical composition of TCKP1 was analyzed by the phenol–sulfuric acid method using glucose as a standard [37]. Sulfate and protein were determined according to the reported method [38,39]. The overall glucuronic acid was detected through the carbazole reaction [40].

### 4.3. Characteration of TCKP1

Monosaccharide composition was measured according to the methods of Chen [36] with a minor modification. First, 10 mg of TCKP1 was hydrolyzed with 4 mL of 2 mol/L trifluoroacetic acid (TFA) in a sealed tube under the protection of nitrogen for 12 h at 105 °C. After being concentrated under reduced pressure, 4 mL methanol was added to completely remove TFA. This procedure was repeated five times. The hydrolyzed product was derivatized with 500 μL of 0.5 mol/L methanol–PMP solution together with 500 μL 0.3 mol/L NaOH at 70 °C for 2 h. After being cooled to room temperature, the reaction mixture was neutralized with 500 μL 0.3 mol/L HCl, extracted three times with chloroform to gain the supernatant. The composition of TCKP1 was performed on HPLC (Agilent Technologies 1260) with a UV detector, using an Extend-C18 column (150 × 4.6 mm i.d., 5 μm, Agilent, USA) for analysis. The elution of the PMP derivatives was performed with a mixture of phosphate buffer (0.05 M, pH = 6.9) with 15% acetonitrile (solvent A) and 40% acetonitrile (solvent B) at a flow rate of 1 mL/min, and the wavelength for UV detection was 250 nm. The binary gradient in HPLC is shown in Table 2. The monosaccharide composition of TCKP1 was based on the corresponding signals and retention times in the chromatographic column compared to standard monosaccharides—glucose (Glc), galactose (Gal), glucosamine (GlcN), mannose (Man), xylose (Xyl), arabinose (Ara), glucuronic acid (GlcA), and galacturonic acid (GalA).

TCKP1 with a concentration of 100 μg/mL was prepared in deionized water. Molecular weight and distribution were detected using ESI-MS with a Triple-TOF mass spectrometer (Triple-TOF 5600+, AB Sciex, city, state, USA) in the mass/charge range of 100 to 40,000 Da.

### 4.4. In Vitro Experiments

The anti-HBV activity assay was performed according to the previous report [41,42] with slight modification. The HepG2.2.15 cell line was obtained from Dr. Ningshao Xia (Xiamen University, China) [43]. The anti-HBV activity of TCKP1 was evaluated on the HepG2.2.15 cell line using lamivudine (3TC) as a positive control. HepG2.2.15 cells were cultured at a density of 2 × 10^5^ cells/mL in DMEM supplemented with 10% (*v*/*v*) of fetal bovine serum, 100 U/mL penicillin, 100 U/mL streptomycin, and 400 μg/mL G418. The cell lines were treated with different concentrations of TCKP1 (12.5, 25, 50, 100, and 200 μg/mL) in triplicate and incubated at 37 °C in 5% CO_2_ atmosphere. Every three days, the levels of HBeAg and HBsAg in the culture medium were measured with the ELISA kit according to the manufacturer’s instructions. After being treated for three days, intracellular and extracellular IFN-α level were evaluated using commercial ELISA kits. After the treatment for six days, the concentration of HBV DNA in the culture supernatants was measured with an HBV DNA extraction and quantitative detection kit (PCR fluorescence probe method) according to the manufacturer’s instructions. The forward primer was 5′-CTGTGCCTTCTCATCTGCCGG-3′. The reverse primer was 5′-TGGTCTCCATGCGACGTGC-3′. The structure of TaqMan probe was (FAM)-CGTGTGCACTTCGCTTCAC CTCTGC (TAMAR)-3′. HBV DNA in samples was detected with a Bio-Rad CFX96 Real-Time PCR Detection System (Hercules, CA, USA). The cycling program was as follows: 50 °C for 2 min, 95 °C for 3 min, and 40 cycles at 95 °C for 10 s and 60 °C for 30 s.

The cytotoxicity of TCKP1 on HepG2.2.15 cells was determined by the CCK-8 method in 96-well plates after treatment for 3 days, and the absorbance was evaluated in a microplate reader at 450 nm after incubating for 2 h at 37 °C.

### 4.5. Animal Experiment Design

The experimental protocols were approved by Ethics Committee for the Management of Laboratory Animals, School of Medicine, Huaqiao University. All animals were treated according to the procedures outlined in the Guide for the Care and Use of Laboratory Animals prepared by the National Academy of Sciences and published by the National Institutes of Health. B6-Tg HBV transgenic mice were purchased from Beijing Vitalstar Biotechnology Co., Ltd. (Beijing, China), containing terminally redundant, 1.28 times length copies of the complete HBV genome, which were created based on the method of microinjection technology of fertilized egg [44]. The HBV-Tg mice were raised in a specific pathogen-free (SPF) barrier environment with 12 h rhythm of light, and free access to standard food and water. Then twenty-five 5-week-old SPF HBV-Tg mice were randomly divided into five groups, including a negative control group (NC, normal HBV transgenic mice treated with PBS), a positive control group (treated with 10 mg/kg·d tenofovir alafenamide, TAF), and TCKP1 low- (50 mg/kg·d), medium- (100 mg/kg·d), and high-dose (200 mg/kg·d) groups (TCKP1-L, TCKP1-M, and TCKP1-H), respectively, five mice for each group. The experimental mice were treated via intraperitoneal injection daily. After treatment for 28 days, all the mice were fasted and only fed with water for 12 h. Then 0.5–1.0 mL blood samples were taken from eyeballs. The mice were sacrificed by cervical dislocation after collecting the blood sample. The livers of all experimental mice were weighed and divided into two parts. A portion from left lobe was fixed in 4% paraformaldehyde tissue fixation solution (Biosharp, Beijing Labgic Technology Co., Led, Beijing, China) for 12 h, and the rest of liver was snap-frozen in liquid nitrogen and then stored at −80 °C prior to analysis.

### 4.6. Serum HBV DNA Extraction and Analysis

The blood samples of experimental mice were centrifugation after standing for 4 h at room temperature and mice serum was collected. HBV DNA in serum was measured with an HBV DNA extraction and quantitative detection kit (PCR fluorescence probe method) according to the manufacturer’s instructions. Samples were amplified and detected with a Bio-Rad CFX96 Real-Time PCR Detection system and the cycling program was as follows: 50 °C for 2 min, 95 °C for 3 min, and 40 cycles at 95 °C for 10 s and 60 °C for 30 s.

### 4.7. Serum HBsAg, HBeAg and Aminotransferase

Serum HBsAg and HBeAg were measured with the ELISA diagnostic kit for HBsAg and HBeAg [45] according to the manufacturer’s instructions. The kit, which is clinically widely used for antibody detection of hepatitis B patients, could accurately detected the inhibiting ability compared with the negative control, rather than quantities of antigens. ALT and AST were assayed using ALT and AST kits (micro method).

### 4.8. Extraction and Analysis of Liver HBV DNA, HBsAg, and HBeAg

Accurately, 20 mg of liver tissue was added to 0.5 mL RIPA buffer and centrifuged after smashing. The subsequent extraction and analysis of HBV DNA, HBsAg, and HBeAg in the supernatant were carried out according to the procedure with serum above.

### 4.9. Extraction and Analysis of Liver HBV RNA

For quantitative RT-PCR, approximately 10 mg of liver tissue was obtained from mice for extraction of total RNA with Cell/Tissue Total RNA Isolation Mini Kit according to the manufacturer’s instructions. Then RNA samples were reverse-transcribed at 50 °C for 15 min using cDNA synthesis kit (HiScript II Q RT Super Mix for qPCR). cDNA was detected using HBV DNA extraction and quantitative detection kit (PCR fluorescence probe method). The cycling program was as follows: 95 °C for 3 min and 40 cycles at 95 °C for 10 s and 60 °C for 30 s.

### 4.10. Histopathological Examination of the Livers

Liver issues were sectioned and stained with hematoxylin–eosin (HE) for the histological analysis [46]. Fragments of mouse liver were fixed in 4% paraformaldehyde tissue fixation solution for 12 h, dehydrated with ethanol solution from 50 to 100%, transparentize with xylene, embedded in paraffin, cut into 2 μm sections, and stained with HE dyes for photomicroscopic observation. All specimens were evaluated on a blinded basis.

### 4.11. Determination of IFN-γ, IL-12, and IL-4 in Serum of Mice

Extraction of liver tissue of HBV-Tg mice was carried out as described above. Levels of IFN-γ, IL-12, and IL-4 in serum were evaluated using commercial ELISA kits according to the corresponding operating instructions.

### 4.12. Statistical Analysis

All experimental data were processed in Microsoft Office Excel 2013 (MS) to obtain descriptive statistics, and were expressed as the mean ± standard deviation (X ± SD). One-way analysis of variance (ANOVA) followed by Dunnett’s multiple comparisons test was performed with the SPSS 19.0 statistical package (SPSS Inc., Chicago, IL, USA), with *p* < 0.05 considered significant and 50% inhibition concentration (IC_50_) and 50% cytotoxicity concentration (CC_50_) were calculated using regression analysis, Probit in SPSS 19.0.

## Figures and Tables

**Table 1:** Monosaccharide composition of TCKP1.

Monosaccharide	Regression Equation	Peak Area (mAu·s)	Contents (μg)	Molar Ratio
Man	y = 0.0001x + 0.0687	2848.4	0.3535	9
GlcN	y = 0.0002x − 0.1629	4709.2	0.7789	16
GlcA	y = 0.00001x + 0.1597	919.7	0.1689	4
Glc	y = 0.0002x + 0.0511	100,494.0	20.15	512
Gal	y = 0.0001x − 0.0117	3671.6	0.3555	9
Ara	y = 0.0001x − 0.0595	1914.0	0.1319	4

**Table 2:** The binary gradient in HPLC.

Time (Min)	A (%)	B (%)
0	85	15
30	85	15
35	60	40
40	60	40

A: phosphate buffer (0.05 M, pH = 6.9) with 15% acetonitrile. B: phosphate buffer (0.05 M, pH = 6.9) with 40% acetonitrile.

## Data Availability

Not applicable.
